# Scrotal Pearls and Hydrocele: A Unique Case of Scrotal Lithiasis

**DOI:** 10.7759/cureus.79027

**Published:** 2025-02-14

**Authors:** Bhavyadeep Korrapati, Vijayanand Mani, Velmurugan Palaniyandi, Hariharasudhan Sekar, Sriram Krishnamoorthy

**Affiliations:** 1 Urology, Sri Ramachandra Institute of Higher Education and Research, Chennai, IND

**Keywords:** hydrocele, loose bodies, scrotal calculi, scrotal pearl, tunica vaginalis

## Abstract

Scrotal calculi are uncommon, benign stones that typically form within the hydrocele sac, often due to chronic inflammation, minor trauma, or torsion of the appendix testis, and are incidentally detected during imaging or surgery. Although rare, scrotal calculi have been reported to occur in specific groups, such as athletes, who experience repetitive mechanical stress. A 56-year-old diabetic male presented with a two-year history of painless scrotal swelling. The swelling is not associated with any trauma. On physical examination, the swelling transilluminated, and an ultrasound revealed an 8-cm hydrocele with a 1.4-cm free-floating lesion, which was isoechoic to the surrounding tissue. The testes and epididymis appeared normal. Surgical exploration confirmed the presence of the hydrocele and a 1.4-cm, pearly white, free-floating scrotal calculus, which was removed. The tunica vaginalis was excised, everted, and plicated to prevent future recurrence of the hydrocele. While rare, scrotal calculi should be considered when a free-floating lesion is detected in the scrotal sac, especially in association with a hydrocele. Surgery is the treatment of choice if patients are symptomatic and generally have a favorable prognosis. Further studies are needed to clarify the underlying causes of scrotal calculi and their prevalence, particularly in populations subject to repetitive scrotal trauma, such as athletes.

## Introduction

A hydrocele is described as a fluid collection in the tunica vaginalis, a thin sac that surrounds the testicles, resulting in a swelling of the scrotum. An imbalance between the secretion and reabsorption of fluid within the tunica vaginalis causes hydrocele [1]. On ultrasound, a hydrocele appears as an anechoic or clear area surrounding the testicle [1]. Ultrasound also helps determine the size and type of the hydrocele and easily distinguishes it from conditions such as spermatoceles, testicular tumors, and testicular atrophy [2]. Hydrocelectomy is the recommended procedure for patients with hydroceles.

Intrascrotal calculi, sometimes referred to as "scrotal pearls" or "fibrinoid loose bodies," are small, calcified objects that form between the layers of the tunica vaginalis [3]. They are usually harmless and freely mobile. The exact cause remains unclear but may be associated with inflammation and calcification. These calculi have been rarely described in the literature, first by Kickham in 1935 and later in 2008 by Vijayananthan et al., where intrascrotal calculi were noted to be less than one cm in size [4,5].

Scrotal ultrasound is a valuable diagnostic tool for identifying these calculi, as demonstrated in our case, where they were found alongside a large 1.1-cm hydrocele [6].

## Case presentation

**Figure 1:**
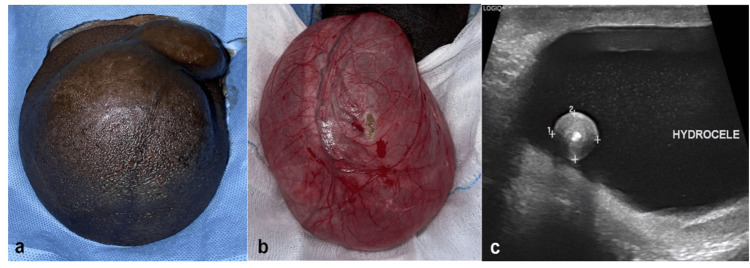
(a) Clinical, (b) intra-operative, (c) and ultrasound finding showing the hydrocele and intra-scrotal loose body. (a) A clinical picture of a right hydrocele of size 8 x 10 cm. (b) The hydrocele after being dissected from the scrotal skin and separated, which is completely cystic in nature. (c) A hydrocele of size 8 cm with a concentric free-floating body in the hydrocele.

A 56-year-old diabetic male presented with a two-year history of scrotal swelling. The swelling was not associated with pain, fever, or trauma. An 8 cm x 4 cm right scrotal swelling was noted on physical examination (Figure 1). The swelling was brilliantly transilluminated, cystic in consistency, and allowed palpation above the mass. Blood investigations revealed no abnormalities. Scrotal ultrasound demonstrated an 8-cm hydrocele with a 1.4-cm concentric, isoechoic, free-floating lesion within the right scrotal sac. Both the testes and the epididymis appeared normal.

Figure 1c shows a concentric isoechoic free-floating lesion with central calcification of size 1.4 cm. The concentric nature indicates the chronicity that gives it a layered appearance. The central calcification suggests a hardened and mineralized core that appears bright and hyperechoic on ultrasound imaging, with possible post-acoustic shadowing. The free-floating nature suggests that the lesion is not attached or adhered to the tunica vaginalis. Figure 1c illustrates a hydrocele of size 8 cm with a free-floating body. The hydrocele is significantly enlarged and indicates a larger fluid accumulation within the scrotal layers. These ultrasound features are representative of a hydrocele with an internal mobile structure with a calcified core that warrants further treatment.

**Figure 2:**
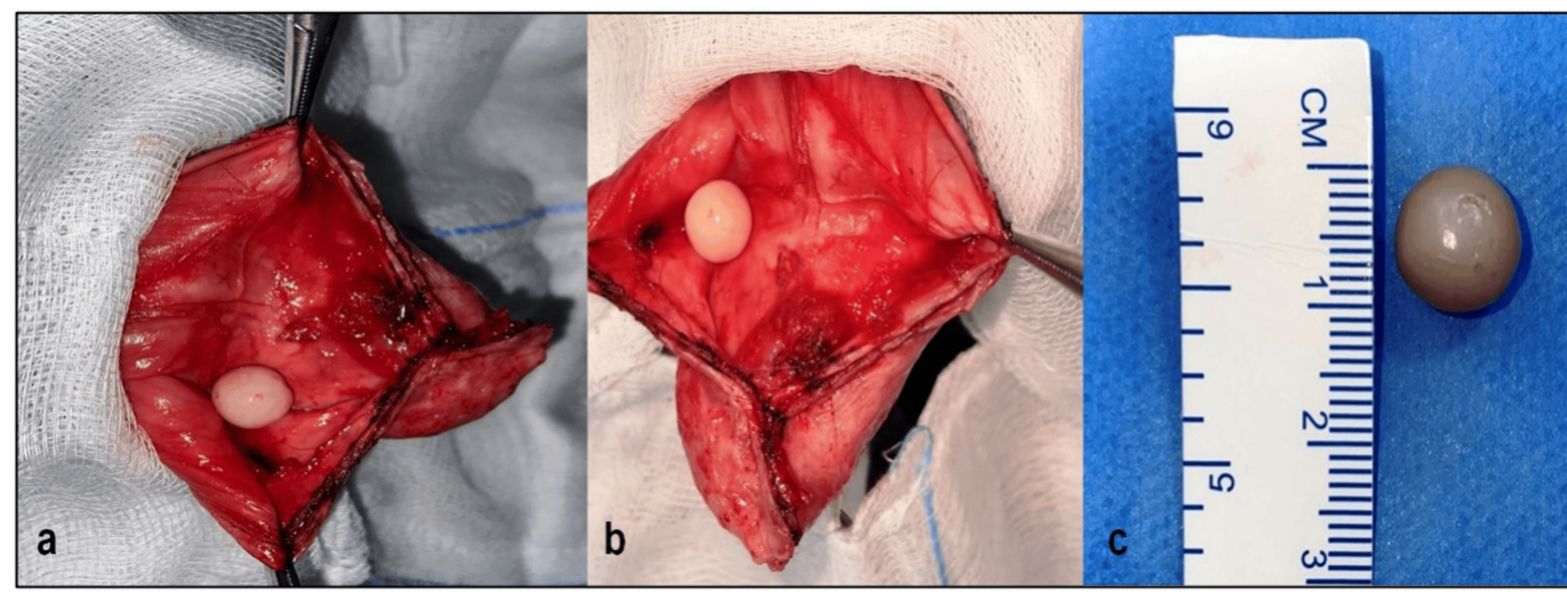
Intraoperative image of the pearl white loose body in the intra-scrotum (a, b, and c) An intra-operative view of the pearl-white loose body found in the hydrocele sac. This loose body appears clearly defined, well-circumscribed, round, firm, and around 1.2 cm in size, has a smooth glistening surface, is pearly white in appearance, and contrasts well against the red tissue background.

Under regional anesthesia, in a supine position, a right paramedian incision was made. The layers were dissected to expose the right testis along with the hydrocele. The tunica vaginalis was incised, and the fluid was suctioned out. A 1.2-cm concentric, pearly white loose body was identified and removed during dissection (Figure 2). Jaboulay procedure was performed, and the tunica vaginalis was excised, everted, and plicated after achieving hemostasis. The skin was then closed in layers.

## Discussion

Scrotal pearls are benign and incidental findings, and their clinical relevance lies in the potential to mimic more serious conditions. Usually, they are asymptomatic. Kickham, in 1935, described scrotal calculi as "calcified hydroceles mimicking tumors." These structures are typically round, pearly white, and rubbery, with a core composed of hydroxyapatite [4]. A distinctive feature of scrotal calculi is their ability to retain water content after drying, provided they are rehydrated [7]. They are generally small, measuring less than 1.5 cm, with the majority around 0.4 cm. The largest recorded scrotal calculus measured 8.7 cm × 7.2 cm × 6.5 cm [7].

The etiology of scrotal calculi is not well understood, but it is hypothesized to involve inflammation of the tunica vaginalis or torsion of the appendix testis or epididymis. Inflammation may lead to tissue damage, fibrosis, and the deposition of substances such as calcium, fibrin, and cholesterol, culminating in the formation of calculi. Another proposed mechanism is the calcification of remnants following torsion; however, this explanation was not applicable in the present case due to the absence of testicular damage [8].

Although considered rare, scrotal calculi appear to be more prevalent in specific populations. For instance, Frauscher et al. reported that 81% of extreme mountain bikers exhibited scrotal calculi, likely due to the repetitive shock and vibration experienced during cycling on rough terrain [7]. This increased prevalence often co-occurs with other scrotal abnormalities such as torsion of testicular appendix and torsion testis.

The prevalence of scrotal calculi in the general population is less well-defined, with reported rates ranging from 1-2% to as high as 2.65% in some studies. Most cases are identified incidentally during surgical procedures or imaging studies, although some patients may present with pain or discomfort [9].

Sonography, particularly with high-resolution transducers, is the diagnostic modality of choice for scrotal calculi. These appear on ultrasound as bright, echogenic foci with posterior acoustic shadowing. Their mobility within a hydrocele helps distinguish them from other types of scrotal pathology [10].

While scrotal calculi are generally benign and rare, further research is warranted to elucidate their pathogenesis and establish a more accurate estimate of their incidence in the general population [11].

Learning points

Scrotal calculi, or "scrotal pearls," are rare benign structures often found within hydroceles. They are typically small, pearly white, and calcified, and their etiology is unclear. Scrotal calculi are commonly associated with hydroceles and are thought to form due to chronic inflammation, trauma, or torsion. High-resolution ultrasound is the gold standard for detecting scrotal calculi. It can identify their bright echogenic appearance, mobility within the hydrocele, and posterior shadowing. Surgical excision of the calculus, combined with hydrocele repair, is the treatment of choice for symptomatic cases. With proper surgical management, most cases have a favorable outcome. Certain populations, such as extreme mountain bikers, may exhibit a higher prevalence of scrotal calculi due to repetitive mechanical stress and undetected trauma to the scrotum.

## Conclusions

Scrotal calculi, though rare, are benign lesions often associated with hydroceles and should be considered in the differential diagnosis of free-floating scrotal lesions. High-resolution ultrasound is an essential diagnostic tool for detecting scrotal calculi and differentiating them from other scrotal pathologies. Surgical removal, combined with hydrocele repair, is the recommended treatment for symptomatic cases, with generally favorable outcomes. Further studies are needed to clarify the pathogenesis, prevalence, and contributing factors, especially in populations prone to repetitive scrotal trauma.
